# Systems Pharmacology–Based Dissection of Anti-Cancer Mechanism of Traditional Chinese Herb *Saussurea involucrata*

**DOI:** 10.3389/fphar.2021.678203

**Published:** 2021-06-25

**Authors:** Qian Zhang, Lanyu He, Qingqing Jiang, Hongqing Zhu, Dehua Kong, Hua Zhang, Zhiqiang Cheng, Hongtao Deng, Yaxin Zheng, Xue Ying

**Affiliations:** ^1^ School of Pharmacy/Key Laboratory of Xinjiang Phytomedicine Resources and Utilization, Ministry of Education, Shihezi University, Xinjiang, China; ^2^ School of Pharmaceutial Sciences/Key Laboratory of Sichuan Province for Specific Structure of Small Molecule Drugs, Chengdu Medical College, Chengdu, China; ^3^ Department of Pharmacology and Molecular Sciences, Johns Hopkins School of Medicine, Baltimore, MD, United States

**Keywords:** systems pharmacology, *Saussurea involucrata*, cancer, action mechanism, active compounds

## Abstract

Cancer has the highest mortality in humans worldwide, and the development of effective drugs remains a key issue. Traditional Chinese medicine *Saussurea involucrata* (SI) exhibits a series of effects, such as anti-cancer, but the action mechanisms are still unclear. Here, systems pharmacology was applied to reveal its anti-cancer mechanism. First, we screened the active compounds of SI. Then, the compound–target network, target–disease network, and target–pathway network were constructed. DAVID was applied for GOBP analysis and KEGG pathway enrichment analysis on cancer-related targets. Seven potential compounds and 187 targets were identified. The target–disease classification network showed that compounds mainly regulated proteins related to cancer, nervous system diseases, and cardiovascular system diseases. Also, SI anti-tumor effect mainly associated with the regulation of NO production, angiogenesis, MAPK, and PKB from GOBP enrichment. Additionally, KEGG pathway enrichment indicated that targets involved in anti-inflammatory action, inhibiting angiogenesis and anti-proliferation or inducing apoptosis. Experimental validation showed that four active compounds could inhibit cell proliferation and promote apoptosis in A549 (except for kaempferol), PC-3, and C6 cells. This study not only provides experimental evidence for further research on SI in cancer treatment but also promotes the development of potential drugs of SI in modern medicine.

## Introduction

Cancer has become one of the major diseases that threaten human health globally (Siegel et al., 2020) and brought a huge economic burden to the world until now. According to the statistics, lung cancer is a leading cause of cancer death with the highest morbidity and on the rise gradually. Breast cancer, prostate cancer, and colorectal cancer are next only to lung cancer (Bray et al., 2018). Currently, conventional cancer treatment is the combination of surgery, radiotherapy, and chemotherapy. As one of the main methods, chemotherapy is indispensable in cancer treatment, but the serious adverse effects of chemotherapeutic drugs limit its further development and application (Zhai et al., 2019). Facing such situations, it is urgently necessary to explore effective drugs and study new therapies. In recent years, traditional Chinese medicine (TCM) is rich in various chemical components and can regulate multiple cancer-related signaling pathways and targets. Besides, it has the advantages of alleviating side effects, providing high safety, and prolonging the survival time of patients (Liu Y. T. et al., 2019; Ma et al., 2019). Consequently, TCM plays an increasingly important role in the process of new anti-cancer drug research. And it is increasingly beneficial to apply TCM for cancer treatment.

*Saussurea involucrata* Kar. et Kir. (SI) is a perennial herbaceous plant that is distributed in high altitude areas of Xinjiang, China (Guo et al., 2019). In traditional Chinese medicine theory, SI has the functions of dispersing cold and removing dampness, activating blood circulation to promote menstruation, strengthening the bones and muscles, warming the kidney, restoring yang, etc. SI is already used for the symptoms of cough due to lung cold, wind–cold–dampness arthralgia, rheumatoid arthritis, and irregular menstruation (Gong et al., 2020b). In modern pharmacology, SI has multiple pharmacological effects such as anti-oxidant, anti-fatigue, analgesic, anti-inflammatory (Wang et al., 2018), and anti-tumor (breast cancer, prostate cancer, etc.) (Gong et al., 2020a), most of which are closely related to its traditional usage. Recently, researchers turned their attention to the anti-tumor effect of SI. However, there are few studies on the connection between specific chemical components and anti-tumor activity. The relationship among active components, potential targets, and related pathways of SI has not been systematically explored.

Confronting with such issues, it is particularly needful to investigate the action mechanism of SI by systems pharmacology. Systems pharmacology (Chen et al., 2018; LUO et al., 2018) is an emerging discipline that can be used as a powerful method for the systematic research on SI. And systems pharmacology breaks the traditional framework of “one drug, one target, one disease” and integrates multi-disciplinary technology and content such as system biology, computational biology, and network topology. Meanwhile, systems pharmacology constructs a multi-level network and systematically explores the relationship between drugs and diseases from an overall perspective. For this reason, it is of great significance to clarify the complex mechanism of traditional Chinese medicine (Zheng et al., 2016).

**FIGURE 1:**
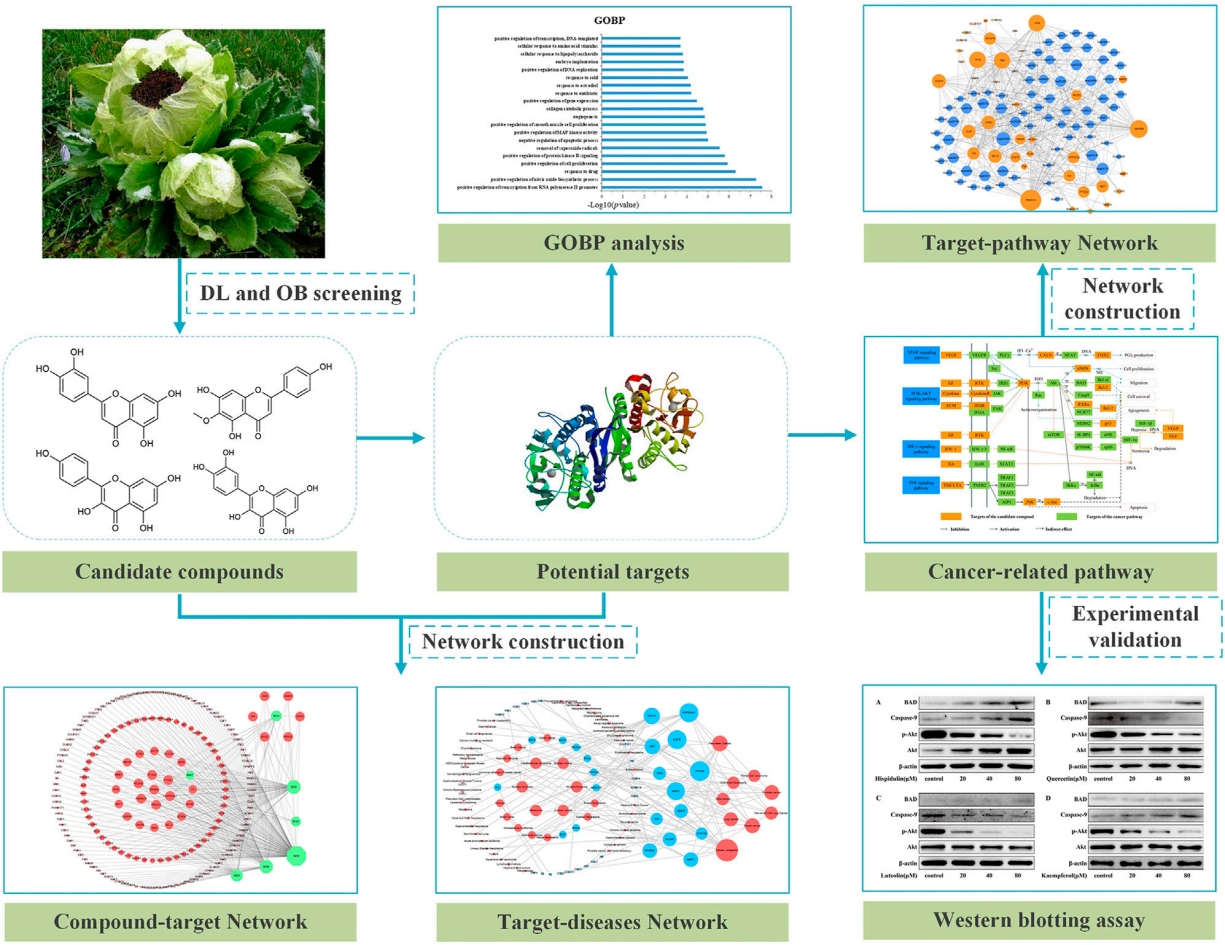
Workflow of systems pharmacology to dissect the anti-tumor mechanism of *Saussurea involucrata.*

In this study, active compounds of SI were screened by systems pharmacology. Then, the compound–target network, target–disease network, and target–pathway network were constructed through the data obtained above. Furthermore, Gene Ontology analysis and KEGG pathway enrichment analysis were applied to analyze the anti-cancer molecular mechanism of SI molecules. Finally, *in vitro* experimental validation proved the regulation of targets by several important compounds (as shown in Figure 1).

## Materials and Methods

### The Screening of Active Compounds of *Saussurea involucrata*

In this study, the Traditional Chinese Medicine Systems Pharmacology Database (TCMSP, http://lsp.nwu.edu.cn/tcmsp.php) was employed to summarize the chemical constituents of *Saussurea involucrata*. Then, drug likeness (DL) and oral bioavailability (OB) were adopted for obtaining active compounds with the keywords of “*Saussurea involucrata*.”

Drug likeness (DL) refers to a molecule that contains specific functional groups and/or has physical characteristics consistent with most of the drugs. In the early stages of drug development, DL was widely used to screen leading compounds (Hu et al., 2018). In order to calculate the drug likeness of these compounds, the Tanimoto coefficient and molecular descriptors were utilized in this database. The formula is as follows:

$$T\left(X,\;Y\right)=\frac{x\cdot y}{{\left|x\right|}^{2}+{\left|y\right|}^{2}-x\cdot y},$$ (1)

where *x* is the molecular descriptor index of SI that is calculated by Dragon and *y* is defined as the average drug likeness index of all compound descriptors in the DrugBank database. The threshold of the DL value is determined by DrugBank’s average DL value (0.18). Therefore, the active compounds of *Saussurea involucrata* were screened with DL ≥ 0.18 in this study.

Oral bioavailability (OB) is the relative amount of a drug absorbed into the circulatory system through extravascular administration. OB is one of the vital indicators to reflect drug efficacy (Yang et al., 2020). In general, drugs with poor oral bioavailability might not be able to reach the minimum effective concentration to achieve therapeutic effect even if they have a strong effect on pharmacological targets *in vitro* (Aungst, 2017). So, compounds with OB ≥ 30% were utilized for subsequent analysis in this work.

### Construction of Compound–Target Network and Target–Disease Network

After the screening procedure mentioned above, seven active compound–related targets were obtained from the TCMSP. And UniProt (https://www.uniprot.org/) was utilized to convert targets into corresponding genes and defined the species as “human.” In order to further analyze the complex relationship between compounds, targets, and diseases, Cytoscape 3.7.1 (Ou et al., 2020) software was used to construct the active compound–target network (C–T network), target–disease network (T–D network), and target–pathway network (T–P network). The plug-in network analyzer of Cytoscape analyzed the degree, which is a critical parameter of these networks.

### Gene Ontology Analysis and KEGG Pathway Enrichment Analysis

For exploring the related biological processes of potential targets, DAVID 6.8 (the Database for Annotation, Visualization and Integrated Discovery, https://david.ncifcrf.gov/) was used for Gene Ontology analysis and KEGG pathway enrichment analysis. Firstly, the target gene list was input to DAVID 6.8, which defined the species as “*Homo sapiens*.” Then, the target gene was modified to the “office gene symbol.” Each GO term had a *p*-value, and the threshold *p* ≤ 0.05 was set, which indicated that target genes were significantly enriched in this biological process.

### Experimental Validation

*In vitro* experiments were performed to validate the integrated anti-cancer pathway of SI. The cancer target–cancer network displayed that SI is associated with multiple cancers, including pancreatic cancer, lung cancer, prostate cancer, breast cancer, colorectal cancer, and renal cell cancer. In this study, A549 cells, C6 cells, and PC-3 cells were cultured and treated with various concentrations of four monomeric active compounds separately. And four proteins of Akt, p-Akt, caspase-9, and BAD were selected to verify the integrated pathway.

#### Cell Culture

Human non-small-cell lung adenocarcinoma A549 cells (Procell Life Science and Technology Co., Ltd., Wuhan, China) were cultured in an RPMI-1640 medium (Gibco, United States) containing 10% fetal bovine serum (Biological Industries, Israel), 100 μg/ml streptomycin, and 100U/mL penicillin (Gibco, United States). The cells were incubated in a saturated humidity incubator (37°C, 5% CO_2_) (Thermo Fisher Scientific, United States).

Rat glioma C6 cells and human prostate cancer PC-3 cells (Procell Life Science and Technology Co., Ltd.) were cultured in a DMEM/F12 (Gibco) medium complemented with 10% fetal bovine serum, 100 μg/ml streptomycin, and 100 U/mL penicillin and incubated in a saturated humidity incubator.

#### Cell Protein Extraction

In the logarithmic growth phase, A549, C6, and PC-3 cells (5 × 10^5^/well) were seeded in six-well plates (Corning incorporated Costar, United States), at 1.9 ml per well, and continued to be cultured at 37°C, 5% CO_2_ condition. Then, a series of concentrations of luteolin (Chengdu Biopurify Phytochemicals Co., Ltd., Chengdu, China), quercetin (Chengdu Biopurify Phytochemicals Co., Ltd., Chengdu, China), kaempferol (Chengdu Biopurify Phytochemicals Co., Ltd., Chengdu, China), and hispidulin (Tauto Biotech Co., Ltd., Shanghai, China) solutions were prepared, and the final concentration gradient of luteolin, quercetin, kaempferol, and hispidulin in each group was 0, 400, 800, and 1600 μM, respectively. And a solvent control group was set. After the cells were almost full, 100 μL of the above-mentioned drugs luteolin, quercetin, kaempferol, and hispidulin (final concentrations of 0, 20, 40, and 80 μM) was added to the six-well plates, respectively. After being incubated at 37°C, 5% CO_2_ condition for 24 h, the original medium was discarded, and lysis buffer was added to lyse the sample on ice for 30 min, followed by centrifuging in a TGL-16G centrifuge (Eppendorf, Germany) at 4°C and 12000 r/min for 8 min. The concentration of protein was measured by the Quawell Q5000 micro-volume UV-Vis spectrophotometer (Quawell Technology, United States). Tris–glycine running buffer (containing 3.03 g/L Tris, 14.4 g/L glycine, and 1 g/L SDS) and SDS-PAGE loading buffer (Beijing ComWin Biotech Co, Ltd., Beijing, China) were used to equalize the protein concentration of each sample to 5 mg/ml. The loading volume of the sample was 10 μL.

#### Western Blot Analysis

After SDS-PAGE gel electrophoresis, protein bands were transferred to PVDF membranes (Merck Millipore, Germany) in an ice bath. The membranes were blocked in 5% skim milk powder for 1 h at room temperature, washed with TBST four times, and incubated with the primary antibody at 4°C overnight. Then, the membranes were washed with TBST, conjugated with the secondary antibody (1: 10000) at room temperature for 2 h, and then washed with TBST buffer to remove the residual secondary antibody. Akt (Cell Signaling Technology, United States), p-Akt (Cell Signaling Technology), BAD (Abcam, United Kingdom), caspase-9 (Abcam), and β-actin (Beijing Jinqiao Biotechnology Co, Ltd., Beijing, China) were the primary antibodies conjugated with the membranes. The membranes were detected by the SuperSignal ^TM^ West Femto Maximum Sensitivity Substrate (Thermo Fisher Scientific) and imaged by the EC3^TM^ 510 Imaging System (Ultra-Violet Products Ltd., Cambridge, United Kingdom). ImageJ software was used for data analysis to semi-quantitate the expression level of protein.

### Statistical Analyses

Data are expressed as mean ± SD. The significance of results was determined based on one-way analysis of variance using Prism 6 (GraphPad, San Diego, CA, United States). *p* < 0.05 was considered significant. All experiments were repeated at least three times.

## Results

### The Screening of Active Compounds

The 55 compounds of SI were collected from the TCMSP, including flavonoids, alkaloids, and sterols. As a result, seven compounds were screened out with the condition of OB ≥ 30% and DL ≥ 0.18, which were hispidulin, alloisoimperatorin, β-sitosterol, kaempferol, luteolin, flazin, and quercetin. The detailed information on these compounds is shown in Table 1.

**TABLE 1:** Information on candidate compounds in *Saussurea involucrata.*

No.	Compounds	Degree	OB	DL	Structure
M10	Hispidulin	17	30.97	0.27	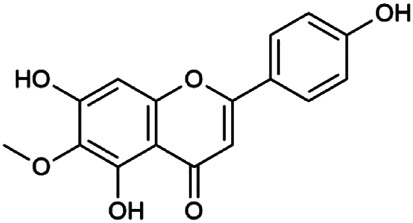
M12	Alloisoimperatorin	6	34.8	0.22	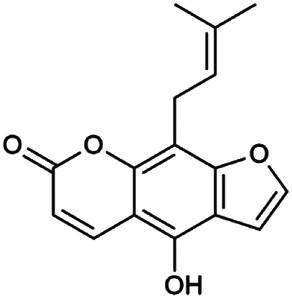
M24	β-Sitosterol	36	36.91	0.75	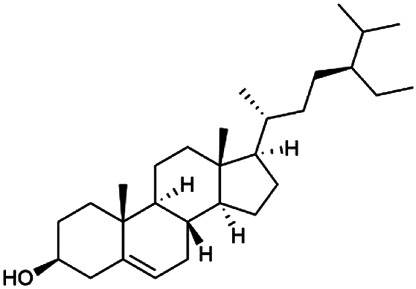
M29	Kaempferol	60	41.88	0.24	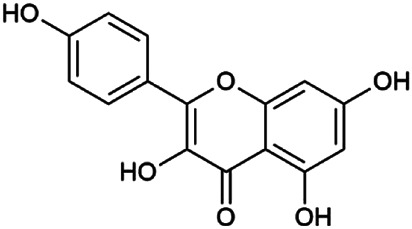
M36	Luteolin	55	36.16	0.25	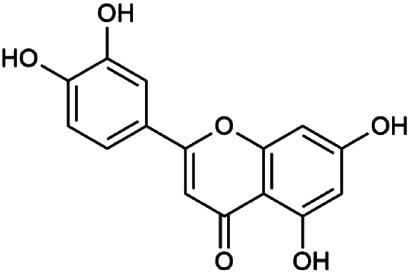
M47	Flazin	3	94.28	0.39	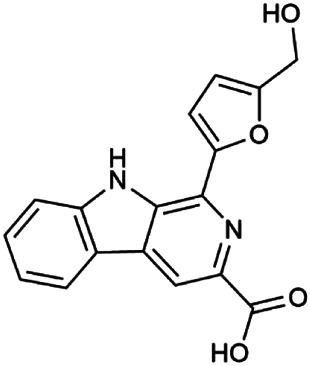
M55	Quercetin	145	46.43	0.28	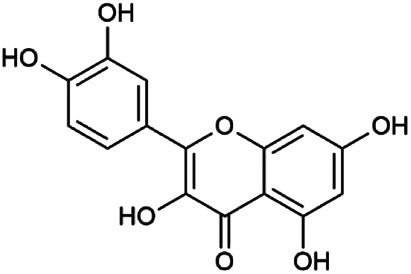

### Network Analysis

**FIGURE 2:**
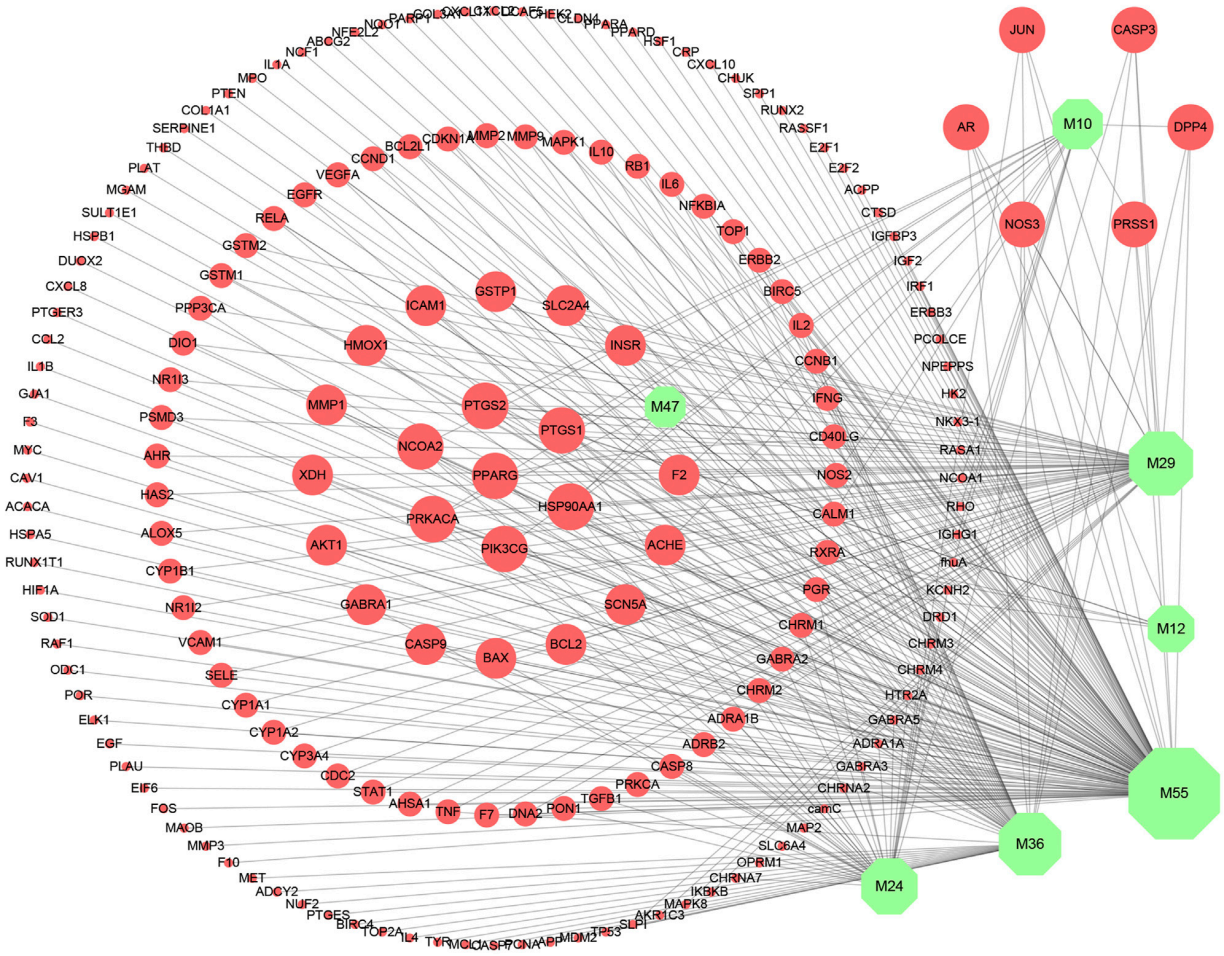
Compound–target network. Green nodes represent compounds from *Saussurea involucrata*, and red nodes represent targets. The node size is proportional to its degree.

In this study, compounds’ targets were obtained from the TCMSP and UniProt was used to query target information (Supplementary Table S1). Then, the C–T network of SI was constructed by Cytoscape 3.7.1 to visualize the interactions between compounds and targets. As shown in Figure 2, nodes represent the active compounds and their corresponding targets of SI, while edges represent the relationship between them. Besides, the degree of the node is defined as the number of edges connected to the node, which is proportional to the importance of the node in the network. The C–T network of candidate compounds consists of 194 nodes and 322 edges. Among them, five compounds showed high degree (degree ≥ 10), indicating that they play a key role in the network. At the same time, each active compound is associated with multiple targets, and targets partly are the same. These suggest the multi-target characteristics of the active ingredients and the potential synergistic effects among compounds.

**FIGURE 3:**
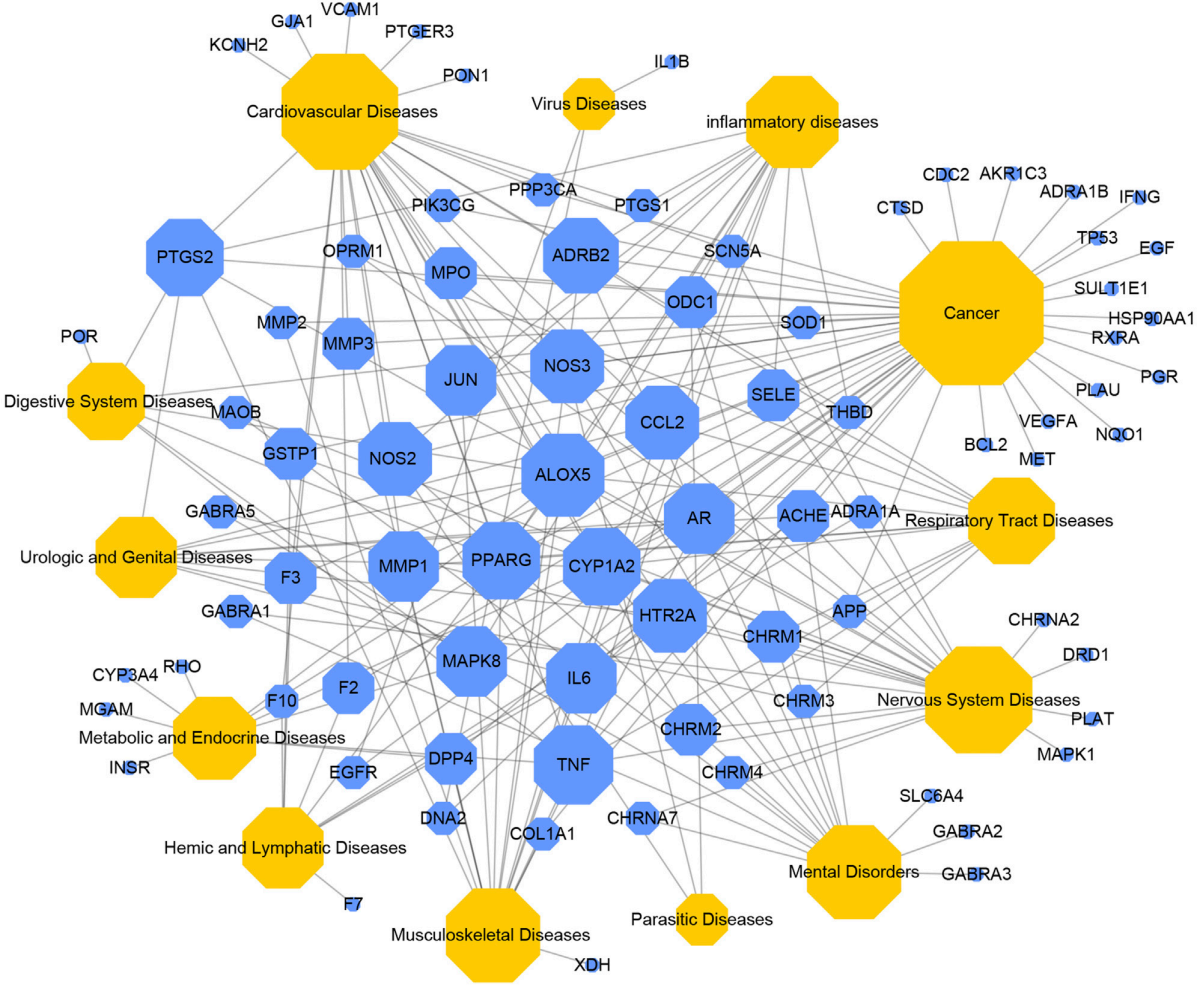
Target–disease network. Blue nodes represent targets, and yellow nodes represent diseases. The node size is proportional to its degree.

**FIGURE 4:**
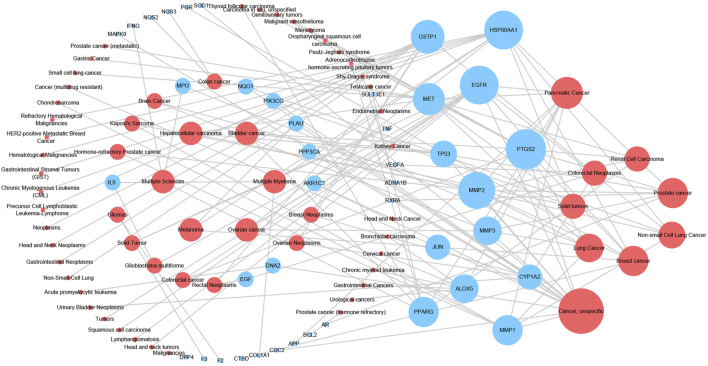
Cancer target–cancer network. Red nodes represent cancer, and blue nodes represent the cancer-related targets. The node size is proportional to its degree.

In order to illustrate the action mechanism of SI, the target–disease classification network was constructed based on 95 nodes and 194 edges. As shown in Figure 3, active compounds mainly regulate the proteins related to cancer, nervous system diseases, and cardiovascular system diseases. Subsequently, the cancer target–cancer network was built to deeply explore the relationship between cancer targets and cancer. As shown in Figure 4, the average degree of the potential cancer target is 3.38. It is supposed that active compounds vitally have positive effects on pancreatic cancer, lung cancer, prostate cancer, and breast cancer. Prostaglandin G/H synthase 2 (PTGS2) exhibits the highest degree and regulates 13 types of cancers, followed by HSP90AA1 (degree = 12), EGFR (degree = 12), and MMP2 (degree = 11). The result displays that active ingredients can exert the anti-cancer cumulative therapeutic effect through multiple targets. Hence, we hypothesize that the anti-tumor effect of SI can be achieved by regulating multiple systems.

### Gene Ontology Analysis and KEGG Pathway Enrichment Analysis

**FIGURE 5:**
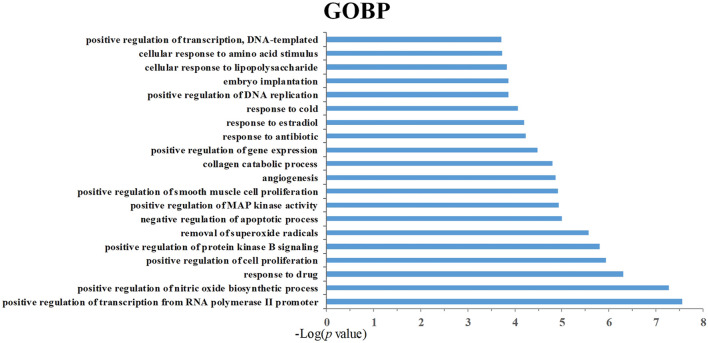
Gene Ontology biological process (GOBP) analysis of target genes. The y-axis shows significantly enriched “biological process” (BP) categories in GO relative to the target genes, and the x-axis shows the enrichment scores of these terms (*p*-value ≤ 0.05).

**FIGURE 6:**
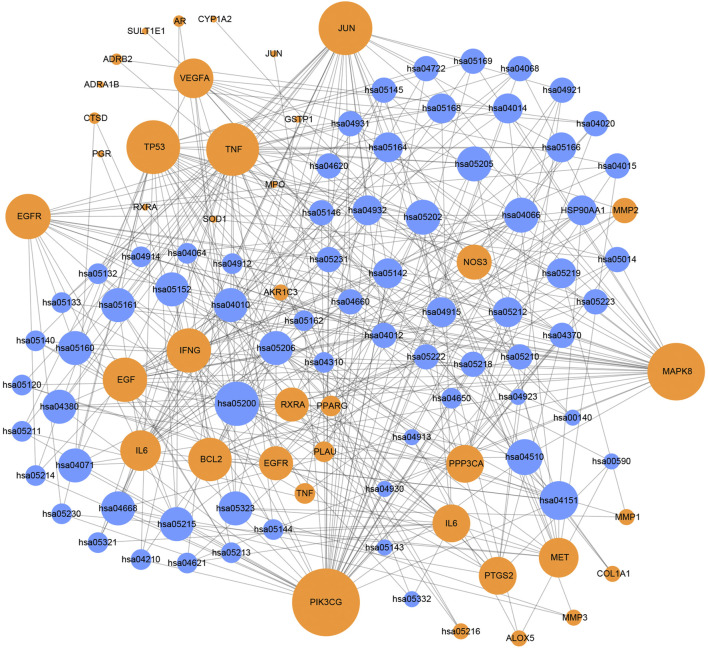
Target–pathway network. Blue nodes represent pathways, and orange nodes represent targets. The node size is proportional to its degree.

**FIGURE 7:**
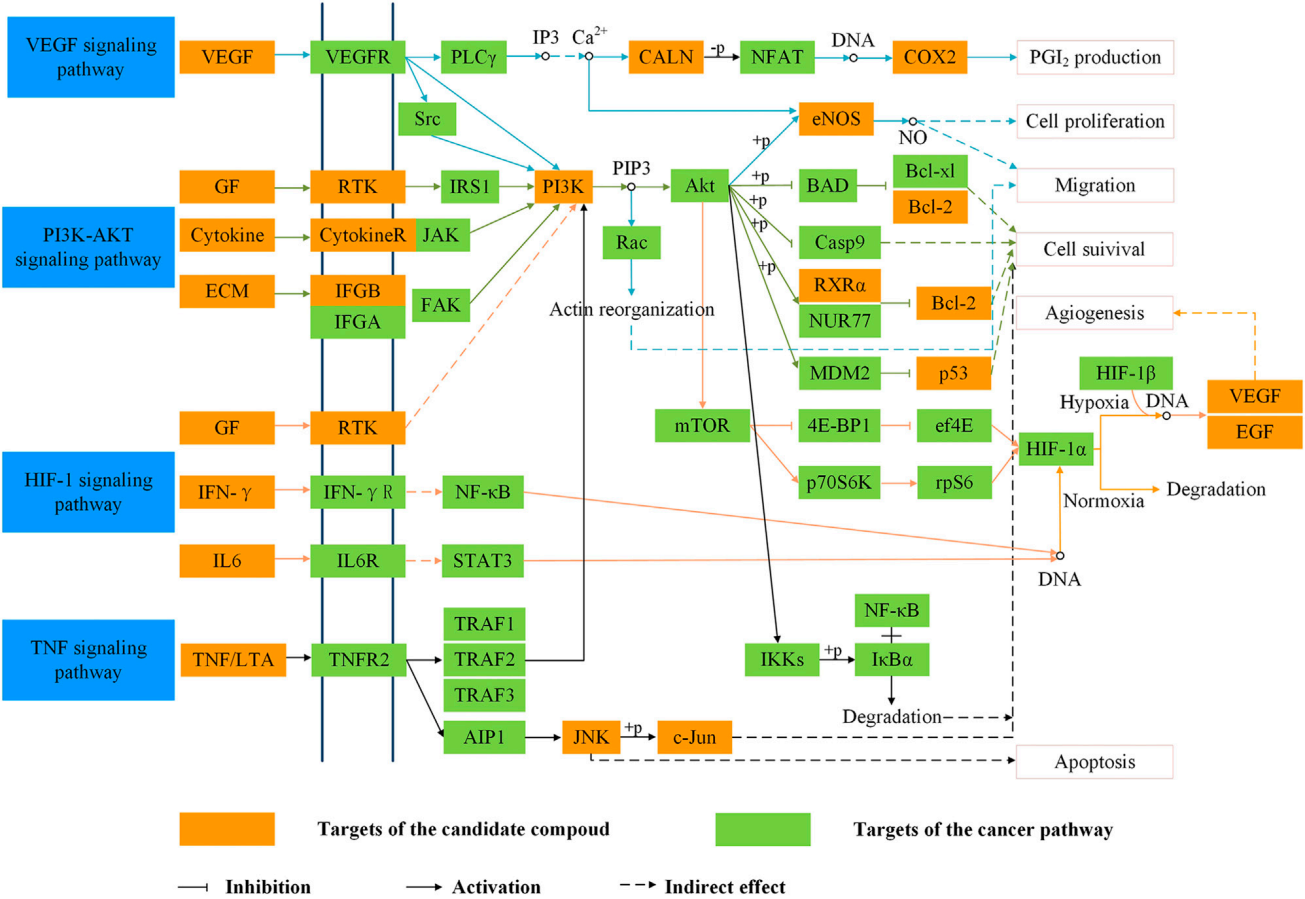
Representative cancer pathway and therapeutic modules of *Saussurea involucrata.* The arrow stands for the activation effect, the t-arrow stands for the inhibition effect, and the dotted line represents indirectly activation active or inhibition active.

In order to analyze the corresponding relationship between target genes and biological processes, 43 candidate cancer genes were mapped to DAVID for GOBP enrichment. A total of 157 important biological processes were obtained, and the top 20 biological processes in terms of enrichment degree were mapped (Figure 5). The results showed that targets were closely related to multiple biological processes, including positive regulation of transcription from RNA polymerase II promoter, positive regulation of nitric oxide biosynthetic process, response to drug, positive regulation of cell proliferation, and positive regulation of protein kinase B signaling. “Positive regulation of nitric oxide biosynthetic process” may play a crucial role in exerting anti-cancer effects. NO is a unique gas chemical molecule that can directly damage cell DNA with high concentration and lead a variety of cancer cells to death (Liu L. et al., 2019; Sinha et al., 2019; Sung et al., 2019). “Positive regulation of MAP kinase activity” and “positive regulation of protein kinase B signaling” are related to cancer proliferation, migration, and invasion, which can be used as one of the potential strategies for cancer treatment (Wen et al., 2019). Tumor growth is usually accompanied by neovascularization, and “angiogenesis” is an important cause of tumor growth and hematogenous metastasis. These biological processes indicate that SI can treat cancer by inhibiting cell proliferation and migration. Meanwhile, the target–pathway network was constructed. Firstly, targets were mapped to DAVID for KEGG pathway enrichment. After that, the T–P network was drawn based on the enrichment results. As shown in Figure 6, the T–P network was composed of 40 targets and 70 pathways (Supplementary Table S2) (110 nodes and 378 edges). The results showed that SI is highly correlated with four pathways, including the HIF-1 signaling pathway (hsa04066), PI3K-Akt signaling pathway (hsa04151), TNF signaling pathway (hsa04668), and VEGF signaling pathway (has04370). As shown in Figure 7, SI can integrate multiple signaling pathways to inhibit cancer proliferation, promote apoptosis, and inhibit angiogenesis.

### *In Vitro* Experimental Validation

In this study, based on the analysis of systemic pharmacology, *Saussurea involucrata* can integrate multiple pathways to play an anti-tumor role. In order to further verify the integrated anti-cancer pathway of *Saussurea involucrata*, we selected three cancer cells and four active compounds with high correlation and most representative. And we also selected the PI3K-Akt pathway, which basically converged with the other three pathways, to carry out *in vitro* verification experiments.

#### A549 Cell Pathway Validation

**FIGURE 8:**
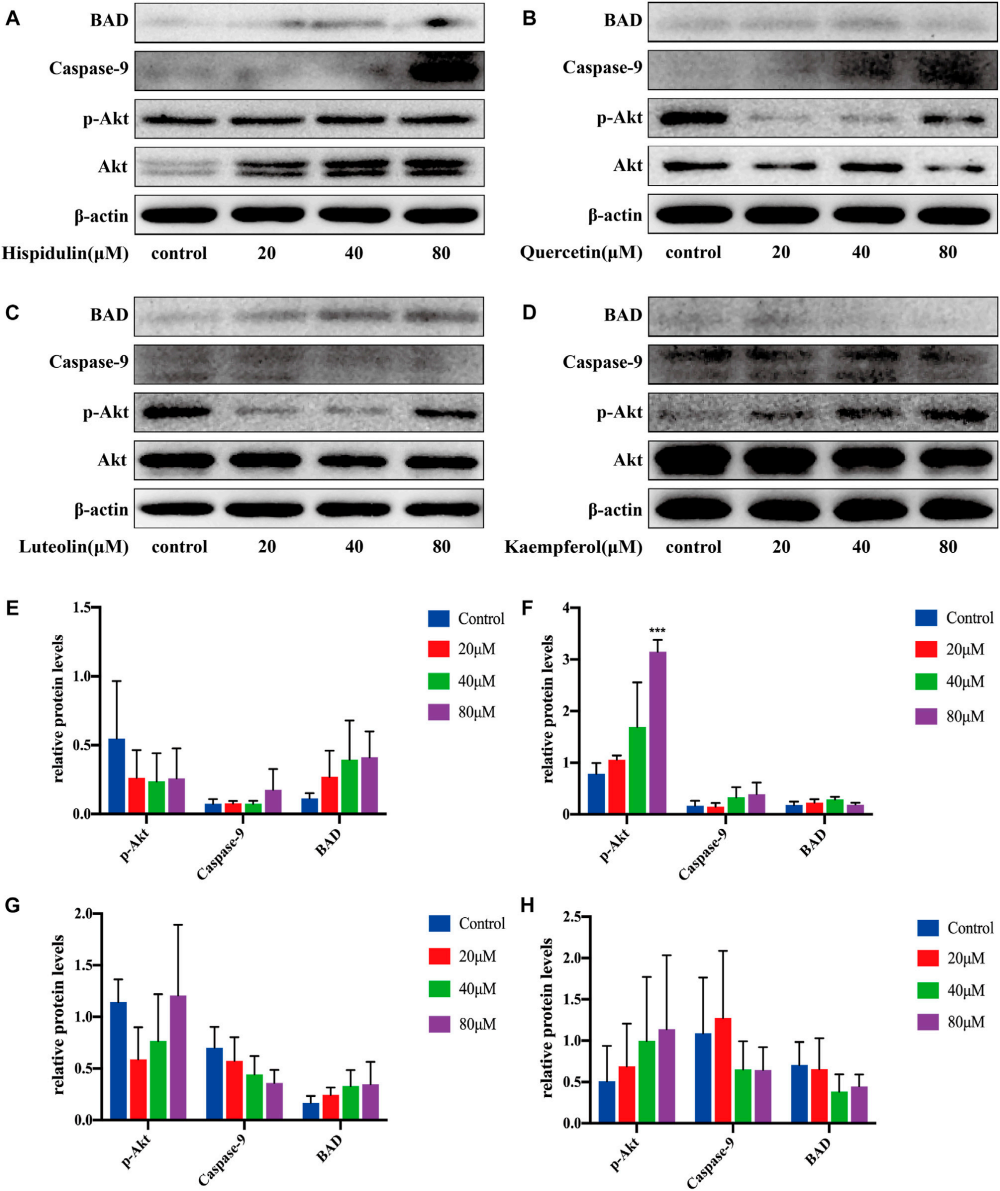
Western blot analysis of Akt, p-Akt, caspase-9, and BAD expressions in A549 cells. **(A)** Cells were treated with or without hispidulin (0, 20, 40, or 80 μM) for 24 h. **(B)** Cells were treated with or without quercetin (0, 20, 40, or 80 μM) for 24 h. **(C)** Cells were treated with or without luteolin (0, 20, 40, or 80 μM) for 24 h. **(D)** Cells were treated with or without kaempferol (0, 20, 40, or 80 μM) for 24 h. **(E)** Quantification data of western blot in group A. **(F)** Quantification data of western blot in group B. **(G)** Quantification data of western blot in group C. **(H)** Quantification data of western blot in group D. Experimental values are expressed as mean ± SD for each group (*n* = 3, ***p < 0.001 with the control group).

After being treated with hispidulin for 24 h, the results of western blot on A549 cells are shown in Figure 8. Hispidulin increased the expression of BAD and caspase-9 and reduced p-Akt expression. Quercetin significantly increased the expression of p-Akt (*p* < 0.001), caspase-9, and BAD in A549 cells. Luteolin inhibited the expression of p-Akt and caspase-9 in A549 cells and increased the expression of BAD. Kaempferol enhanced the expression of p-Akt and suppressed the levels of caspase-9 and BAD. These data showed that hispidulin, luteolin, and quercetin had good anti-tumor effects on A549 cells, while kaempferol was beneficial to the proliferation of A549 cells.

#### PC-3 Cell Pathway Validation

**FIGURE 9:**
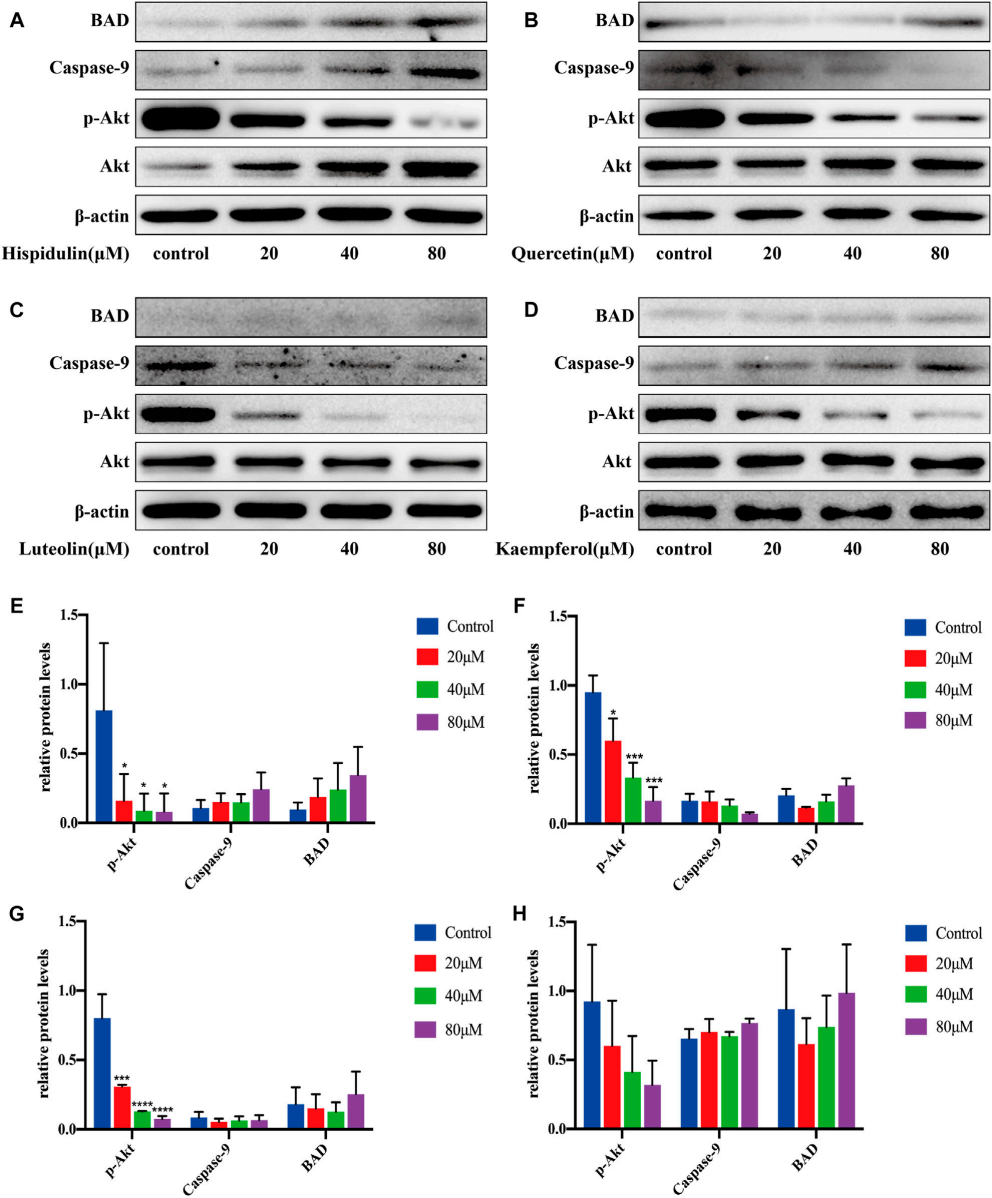
Western blot analysis of Akt, p-Akt, caspase-9, and BAD expressions in PC-3 cells.**(A)** Cells were treated with or without hispidulin (0, 20, 40, or 80 μM) for 24 h. **(B)** Cells were treated with or without quercetin (0, 20, 40, or 80 μM) for 24 h. **(C)** Cells were treated with or without luteolin (0, 20, 40, or 80 μM) for 24 h. **(D)** Cells were treated with or without kaempferol (0, 20, 40, or 80 μM) for 24 h. **(E)** Quantification data of western blot in group A. **(F)** Quantification data of western blot in group B. **(G)** Quantification data of western blot in group C. **(H)** Quantification data of western blot in group D. Experimental values are expressed as mean ± SD for each group (*n* = 3, *p < 0.05,***p < 0.001, and ****p < 0.0001 with the control group).

As shown in Figure 9, hispidulin significantly reduced p-Akt expression (*p* < 0.05) and augmented the expression of BAD and caspase-9. Quercetin significantly decreased the expression of p-Akt (*p* < 0.05, *p* < 0.001, and *p* < 0.001, respectively), increased the expression of BAD, and inhibited the expression of caspase-9 protein. Luteolin significantly downregulated the expression of p-Akt with the increase in drug concentration (*p* < 0.001, *p* < 0.0001, and *p* < 0.0001, respectively), increased the expression of BAD, and decreased caspase-9 expression. Kaempferol decreased the expression of p-Akt and promoted the expression of BAD and caspase-9.

These data showed that, in PC-3 cells, hispidulin, luteolin, quercetin, and kaempferol had good anti-tumor effects.

#### The Validation of C6 Cell Pathway

**FIGURE 10:**
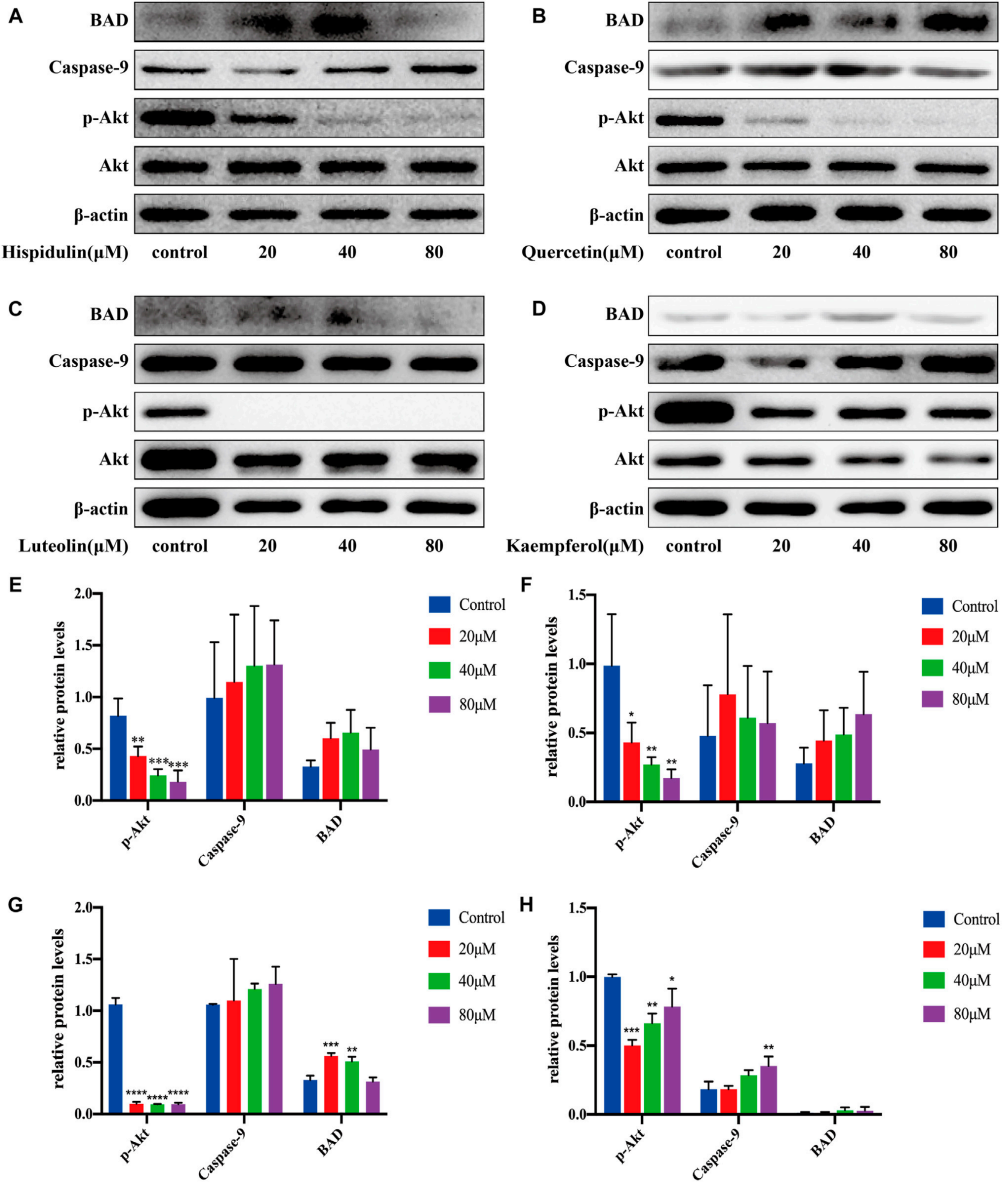
Western blot analysis of Akt, p-Akt, caspase-9, and BAD expressions in C6 cells. **(A)** Cells were treated with or without hispidulin (0, 20, 40, or 80 μM) for 24 h. **(B)** Cells were treated with or without quercetin (0, 20, 40, or 80 μM) for 24 h. **(C)** Cells were treated with or without luteolin (0, 20, 40, or 80 μM) for 24 h. **(D)** Cells were treated with or without kaempferol (0, 20, 40, or 80 μM) for 24 h. **(E)** Quantification data of western blot in group A. **(F)** Quantification data of western blot in group B. **(G)** Quantification data of western blot in group C. **(H)** Quantification data of western blot in group D. Experimental values are expressed as mean ± SD for each group (*n* = 3, *p < 0.05, **p < 0.01, ***p < 0.001, and ****p < 0.0001 with the control group).

As shown in Figure 10, hispidulin significantly reduced p-Akt expression (*p* < 0.001) and increased caspase-9 expression. Quercetin significantly downregulated the expression of p-Akt with the increase in drug concentration (*p* < 0.05, *p* < 0.01, and *p* < 0.01, respectively) and improved the expression of BAD and caspase-9 in C6 cells. Luteolin significantly lowered the expression of p-Akt (*p* < 0.0001) and improved the expression of BAD (*p* < 0.001 and *p* < 0.01, respectively) and caspase-9. Kaempferol significantly decreased the expression of p-Akt (*p* < 0.001, *p* < 0.01, and *p* < 0.05, respectively) and enhanced the expression of BAD and caspase-9 (*p* < 0.01). These data showed that hispidulin, luteolin, quercetin, and kaempferol inhibited the proliferation and promoted the apoptosis of C6 cells.

## Discussion

At present, cancer is increasingly harmful to human health, and the number of deaths still accounts for a large proportion of global deaths. It remains a critical issue for anti-cancer drugs as chemotherapy frequently causes severe side effects. In recent years, traditional Chinese medicine has played a critical role in clinical cancer therapy. SI has various physiological activities such as anti-tumor, but its systemic mechanism of action is still unclear. Therefore, this study employed systems pharmacology to screen active ingredients, analyze networks and pathways by combining with corresponding targets, diseases, and pathways, and explore the mechanism of *Saussurea involucrata* in cancer treatment.

In this study, seven active compounds and 187 related targets were obtained under the conditions of drug likeness and oral bioavailability based on systems pharmacology. The results explained that SI could have therapeutic effect through multiple compounds and multiple targets. In the C–T network, quercetin (M55) displays the highest degree and interacts with 145 target proteins such as HSP90AA1, PTGS2, and PRKACA, which indicates that active ingredients have multi-target characteristics. At the same time, HSP90AA1 is corresponding to seven active compounds, suggesting SI may act on a target through multiple components. Among the seven active ingredients, quercetin can induce apoptosis of various cancer cells, such as colon cancer (Pang et al., 2019), prostate cancer, and gastric cancer (Shang et al., 2018). As a natural flavonoid, kaempferol (M29, degree = 60) has anti-oxidant, anti-inflammatory (Imran et al., 2019), and anti-cancer activities (Mao et al., 2019). In addition, luteolin (M36, degree = 55) is one of the active ingredients of flavonoids in SI, which can control cell apoptosis by regulating the mitochondrial membrane potential (Seydi et al., 2018). Therefore, we speculated that these ingredients are the key ingredients for SI to have an anti-tumor effect.

Furthermore, the target–disease classification network was constructed in order to further analyze the interaction between targets and diseases. And the network indicated that targets mainly regulate proteins related to cancer, nervous system diseases, and cardiovascular system diseases. The cancer target–cancer network indicates that targets have principal effects on pancreatic cancer, lung cancer, and prostate cancer. Besides, SI displays positive effects on PTGS2, HSP90AA1, and other targets to produce anti-inflammatory, anti-proliferative, and anti-angiogenic effects. In detail, PTGS2 is the rate-limiting enzyme in prostaglandin synthesis, which induces the inflammatory environment to promote cancer development and progression (Peng et al., 2018). It is reported that prostaglandins are capable of stimulating cancer proliferation, promoting angiogenesis (Prima et al., 2017), inhibiting cell apoptosis, and enhancing its metastatic function (Kobayashi et al., 2018). Therefore, the effect of pivotal targets on cancer is worthy of further investigation.

The data of GOBP enrichment display that targets are strongly associated with various biological processes such as NO synthesis, MAPK signaling pathway, PKB signaling pathway, and angiogenesis. NO has a variety of cellular biological functions such as vasodilation, inflammation, immunity, and cell vitality regulation, and high concentrations of NO can be toxic to cells (Edholm et al., 2017). While mitogen-activated protein kinase (MAPK) is critical for cell proliferation, survival, and migration and inflammation regulation (Wei et al., 2017), abnormal MAPK signaling plays an important role in cancer occurrence and progression and the determination of response to cancer treatment (Wang et al., 2017). Besides, the abnormal activation of Akt/PKB is also related to human cancer, and PKB is able to enhance the metabolism and viability of cancer cells (Tang et al., 2018). Therefore, PKB signaling pathway inhibition may be a promising strategy for cancer therapy (Shariati and Meric-Bernstam, 2019). In the development of cancer, tumor cells generally secrete high levels of angiogenic factors that contribute to the formation of abnormal blood vessel networks. Due to the fact that blood vessel networks have characteristics of confusion, immaturity, and permeability, resulting in poor blood perfusion of tumors, the abnormal blood perfusion at the tumor site can reduce the efficiency of chemotherapy drug delivery and radiation therapy (Viallard and Larrivée, 2017). Therefore, active components can regulate related targets to impact on cell proliferation and differentiation, participating in inflammation and angiogenesis processes and thus affecting the development and progression of tumors.

The T–P network and integrated SI cancer-related pathway demonstrate that compounds inhibit the proliferation of cancer cells, promote apoptosis, are anti-inflammatory, and inhibit angiogenesis by regulating the HIF-1 signaling pathway, PI3K-Akt signaling pathway, TNF signaling pathway, and VEGF signaling pathway. Moreover, the PI3K-Akt signaling pathway, TNF signaling pathway, and HIF-1 signaling pathway are involved in the cell proliferation and apoptosis module. Activated PI3K can convert PIP2 to PIP3, which induces Akt phosphorylation. Phosphorylated Akt can not only inhibit the activity of BAD, caspase-9, and Bcl-2 to induce cell proliferation and migration but also activate eNOS to generate NO and promote cell proliferation. Hence, SI might have an anti-cancer effect by regulating BAD, caspase-9, Bcl-2, and eNOS. In the inflammatory module, there are the PI3K-Akt signaling pathway and TNF signaling pathway. Inflammation is a physiological response to infection, injury, or chemical stimulation. Chronic inflammation can induce a variety of diseases like cancer. Tumor necrosis factor receptor 1 (TNFR1) activates the transcription factor NF-κB, mediates apoptosis, and acts as an inflammation regulator. The VEGF, PI3K-Akt, TNF, and HIF-1 signaling pathways were involved in the migration module. The VEGF signaling pathway activates PI3K, which can further activate eNOS to generate NO and promote cancer proliferation and metastasis. In addition, the VEGF signaling pathway can activate PLCγ, promote the production of IP3, induce the release of Ca2^+^, then activate COX-2, and increase prostaglandin I_2_ (PGI_2_) production. PGI_2_ has the effects of anti-platelet formation, anti-inflammatory, anti-proliferation, and tumor migration inhibition. The above four pathways basically converge on the PI3K-Akt pathway, so that this pathway is selected as the verification pathway.

In order to further verify the results of systematic pharmacology, we selected the PI3K-Akt signaling pathway for *in vitro* validation experiments. It is well known that the PI3K-Akt signaling pathway is one of the main intracellular signal transduction pathways, which plays a very important role in the pathogenesis of many cancers. Abnormal activation of the PI3K-Akt signaling pathway will lead to abnormal expression of a series of downstream proteins and eventually lead to excessive proliferation of cancer cells. Therefore, the PI3K-Akt signaling pathway is a key target in cancer treatment. Akt, the key protein in the PI3K-Akt signaling pathway, is a serine/threonine kinase that regulates cell survival, proliferation, and apoptosis, angiogenesis, and glucose uptake (Song et al., 2019). Overexpression or overactivation of Akt often leads to abnormal signal transduction and uncontrolled proliferation of related diseases, which is an important feature of many human cancers. When Akt is activated on the cell membrane, it can phosphorylate the serine and threonine in the specific part of the substrate protein, thereby exerting extensive anti-apoptosis effect and promoting cell survival. BAD and caspase-9 are two vital direct downstream substrates of Akt. The Bcl-2 agonist of cell death (BAD) is a pro-apoptotic protein (Wan et al., 2018), which can be inactivated by its phosphorylation in many cancers (Bui et al., 2018), so blocking BAD phosphorylation can promote cell apoptosis. Comparably, caspase-9 is a key protease in the mitochondrial apoptosis pathway, which is located at the top of cascade activation. Activated caspase-9 activates various downstream caspase molecules, causing a series of cascade reactions that induce apoptosis. Our results indicate that hispidulin and quercetin could inhibit p-Akt activation in A549 cells and promote the expression of BAD and caspase-9, respectively. Luteolin is capable of downregulating p-Akt and caspase-9, as well as upregulating BAD simultaneously. But kaempferol enhances the p-Akt level and lowers the expression of caspase-9 and BAD, which is beneficial to A549 cell proliferation and plays an antagonistic role with other drugs. For PC-3 cells, the expression of p-Akt is decreased, while BAD and caspase-9 are increased after being treated with kaempferol and hispidulin. On the contrary, luteolin and quercetin can downregulate p-Akt and caspase-9 and upregulate BAD, indicating that they can inhibit the growth of PC-3 cells and induce their apoptosis. In C6 cells, four compounds could contribute to the phosphorylation of Akt downregulation and BAD and caspase-9 upregulation, manifesting that these compounds play a synergistic role in inhibiting cell proliferation and promoting apoptosis. Consequently, the active compounds in SI can adjust the integrated cancer pathway proteins to display an anti-tumor effect.

In summary, systems pharmacology provides a new and effective method for SI anti-tumor mechanism research in this work. *In vitro* experimental validation manifests that four active ingredients can act on the same target in A549 cells, PC-3 cells, and C6 cells, which proves that the anti-tumor effect of SI has the properties of multiple components. Meanwhile, there are synergistic or antagonistic pharmacological effects among four active ingredients in the same type of cancer, but the strength of anti-tumor effect may be different for unlike tumors. Further experiments are needed to investigate the regulation of this pathway with the complex mixed system of SI in the future.

## Conclusion

In this study, systems pharmacology was used to explore the mechanism of SI in cancer treatment. Firstly, we screened SI active compounds. Secondly, the C–T network, T–D network, and T–P network were constructed. Finally, target enrichment was applied to analyze the relevant targets. It is revealed that quercetin, luteolin, hispidulin, and kaempferol can inhibit the PI3K-Akt pathway, which is closely related to the anti-tumor property of *Saussurea involucrata*. As a result, *Saussurea involucrata* shows therapeutic effect through multiple components acting on the same target. Our study provides a basis for the research and development of novel anti-cancer drugs.

## Data Availability

The original contributions presented in the study are included in the article/Supplementary Material, and further inquiries can be directed to the corresponding authors.

## Abbreviations

DAVID, the Database for Annotation, Visualization and Integrated Discovery; DL, drug likeness; GO, Gene Ontology; KEGG, Kyoto Encyclopedia of Genes and Genomes; OB, oral bioavailability; PTGS2, prostaglandin G/H synthase 2; PGI2, prostaglandin I2; SI, *Saussurea involucrata*; TCM, traditional Chinese medicine; TCMSP, Traditional Chinese Medicine Systems Pharmacology Database; TNFR1, tumor necrosis factor receptor 1.
