# Supplementary material for: Systems Pharmacology–Based Dissection of Anti-Cancer Mechanism of Traditional Chinese Herb Saussurea involucrata
Source: Front Pharmacol. 2021 Jun 25;12:678203. doi: 10.3389/fphar.2021.678203 (PMC8267469; doi:10.3389/fphar.2021.678203)
Supplement: Supplementary file 1 [file Table1.DOCX]

**Supplementary Material(Table S1)**

**Table S1:** The informations of compound-related targets in *Saussurea involucrata.*

| Uniprot ID | Protein names | Gene names | Organism |
| --- | --- | --- | --- |
| P35354 | Prostaglandin G/H synthase 2 | PTGS2 | Homo sapiens (Human) |
| P27487 | Dipeptidyl peptidase 4 | DPP4 | Homo sapiens (Human) |
| P07900 | Heat shock protein HSP 90-alpha | HSP90AA1 | Homo sapiens (Human) |
| P48736 | Phosphatidylinositol 4,5-bisphosphate 3-kinase catalytic subunit gamma isoform | PIK3CG | Homo sapiens (Human) |
| P00734 | Prothrombin | F2 | Homo sapiens (Human) |
| P29474 | Nitric oxide synthase | NOS3 | Homo sapiens (Human) |
| P10275 | Androgen receptor | AR | Homo sapiens (Human) |
| P06401 | Progesterone receptor | PGR | Homo sapiens (Human) |
| P35368 | Alpha-1B adrenergic receptor | ADRA1B | Homo sapiens (Human) |
| P07550 | Beta-2 adrenergic receptor | ADRB2 | Homo sapiens (Human) |
| P10415 | Apoptosis regulator | BCL2 | Homo sapiens (Human) |
| P05412 | Transcription factor AP-1 | JUN | Homo sapiens (Human) |
| P37231 | Peroxisome proliferator-activated receptor gamma | PPARG | Homo sapiens (Human) |
| P35228 | Nitric oxide synthase, inducible | NOS2 | Homo sapiens (Human) |
| P51530 | DNA replication ATP-dependent helicase/nuclease | DNA2 | Homo sapiens (Human) |
| P01375 | Tumor necrosis factor | TNF | Homo sapiens (Human) |
| P45983 | Mitogen-activated protein kinase 8 | MAPK8 | Homo sapiens (Human) |
| P03956 | Interstitial collagenase | MMP1 | Homo sapiens (Human) |
| P05177 | Cytochrome P450 1A2 | CYP1A2 | Homo sapiens (Human) |
| P09917 | Arachidonate 5-lipoxygenase | ALOX5 | Homo sapiens (Human) |
| P09211 | Glutathione S-transferase P | GSTP1 | Homo sapiens (Human) |
| P42330 | Aldo-keto reductase family 1 member C3 | AKR1C3 | Homo sapiens (Human) |
| Q08209 | Serine/threonine-protein phosphatase 2B catalytic subunit alpha isoform | PPP3CA | Homo sapiens (Human) |
| P00533 | Epidermal growth factor receptor | EGFR | Homo sapiens (Human) |
| P08253 | 72 kDa type IV collagenase | MMP2 | Homo sapiens (Human) |
| P05231 | Interleukin-6 | IL6 | Homo sapiens (Human) |
| P04637 | Cellular tumor antigen p53 | P53 | Homo sapiens (Human) |
| P05067 | Amyloid beta A4 protein | APP | Homo sapiens (Human) |
| P08581 | Hepatocyte growth factor receptor | MET | Homo sapiens (Human) |
| P08254 | Stromelysin-1 | MMP3 | Homo sapiens (Human) |
| P15692 | Vascular endothelial growth factor A | VEGFA | Homo sapiens (Human) |
| P00749 | Urokinase-type plasminogen activator (U-plasminogen activator) | PLAU | Homo sapiens (Human) |
| P01133 | Pro-epidermal growth factor | EGF | Homo sapiens (Human) |
| P00441 | Superoxide dismutase [Cu-Zn] | SOD1 | Homo sapiens (Human) |
| P06493 | Cyclin-dependent kinase 1 | CDK1 | Homo sapiens (Human) |
| P13726 | Tissue factor | F3 | Homo sapiens (Human) |
| P49888 | Estrogen sulfotransferase | SULT1E1 | Homo sapiens (Human) |
| P02452 | Collagen alpha-1(I) chain | COL1A1 | Homo sapiens (Human) |
| P01579 | Interferon gamma | IFNG | Homo sapiens (Human) |
| P05164 | Myeloperoxidase | MPO | Homo sapiens (Human) |
| P15559 | NAD(P)H dehydrogenase [quinone] 1 | NQO1 | Homo sapiens (Human) |
| P07339 | Cathepsin D | CTSD | Homo sapiens (Human) |
| P19793 | Retinoic acid receptor RXR-alpha | RXRA | Homo sapiens (Human) |
