# Supplementary material for: Systems Pharmacology–Based Dissection of Anti-Cancer Mechanism of Traditional Chinese Herb Saussurea involucrata
Source: Front Pharmacol. 2021 Jun 25;12:678203. doi: 10.3389/fphar.2021.678203 (PMC8267469; doi:10.3389/fphar.2021.678203)
Supplement: Supplementary file 2 [file Table2.DOCX]

**Supplementary Material(Table S2)**

**Table S2:** The pathway of target enrichment.

| KEGG_ID | Term | Degree |
| --- | --- | --- |
| hsa05200 | Pathways in cancer | 18 |
| hsa04066 | HIF-1 signaling pathway | 8 |
| hsa04151 | PI3K-Akt signaling pathway | 12 |
| hsa05219 | Bladder cancer | 6 |
| hsa05323 | Rheumatoid arthritis | 7 |
| hsa05215 | Prostate cancer | 7 |
| hsa04510 | Focal adhesion | 9 |
| hsa04668 | TNF signaling pathway | 7 |
| hsa05212 | Pancreatic cancer | 6 |
| hsa05202 | Transcriptional misregulation in cancer | 8 |
| hsa04071 | Sphingolipid signaling pathway | 7 |
| hsa04380 | Osteoclast differentiation | 7 |
| hsa05160 | Hepatitis C | 7 |
| hsa05205 | Proteoglycans in cancer | 8 |
| hsa05161 | Hepatitis B | 7 |
| hsa05014 | Amyotrophic lateral sclerosis | 5 |
| hsa04915 | Estrogen signaling pathway | 6 |
| hsa05223 | Non-small cell lung cancer | 5 |
| hsa05142 | Chagas disease (American trypanosomiasis) | 6 |
| hsa04370 | VEGF signaling pathway | 5 |
| hsa05210 | Colorectal cancer | 5 |
| hsa05152 | Tuberculosis | 7 |
| hsa05218 | Melanoma | 5 |
| hsa05222 | Small cell lung cancer | 5 |
| hsa04012 | ErbB signaling pathway | 5 |
| hsa04932 | Non-alcoholic fatty liver disease | 6 |
| hsa04660 | T cell receptor signaling pathway | 5 |
| hsa05231 | Choline metabolism in cancer | 5 |
| hsa05144 | Malaria | 4 |
| hsa04010 | MAPK signaling pathway | 7 |
| hsa05164 | Influenza A | 6 |
| hsa05146 | Amoebiasis | 5 |
| hsa04620 | Toll-like receptor signaling pathway | 5 |
| hsa05213 | Endometrial cancer | 4 |
| hsa04931 | Insulin resistance | 5 |
| hsa05145 | Toxoplasmosis | 5 |
| hsa05168 | Herpes simplex infection | 6 |
| hsa04621 | NOD-like receptor signaling pathway | 4 |
| hsa04722 | Neurotrophin signaling pathway | 5 |
| hsa05169 | Epstein-Barr virus infection | 5 |
| hsa04210 | Apoptosis | 4 |
| hsa05206 | MicroRNAs in cancer | 7 |
| hsa05321 | Inflammatory bowel disease | 4 |
| hsa05230 | Central carbon metabolism in cancer | 4 |
| hsa05214 | Glioma | 4 |
| hsa05211 | Renal cell carcinoma | 4 |
| hsa05120 | Epithelial cell signaling in Helicobacter pylori infection | 4 |
| hsa04068 | FoxO signaling pathway | 5 |
| hsa05140 | Leishmaniasis | 4 |
| hsa05133 | Pertussis | 4 |
| hsa04014 | Ras signaling pathway | 6 |
| hsa04921 | Oxytocin signaling pathway | 5 |
| hsa05216 | Thyroid cancer | 3 |
| hsa05132 | Salmonella infection | 4 |
| hsa04914 | Progesterone-mediated oocyte maturation | 4 |
| hsa04064 | NF-kappa B signaling pathway | 4 |
| hsa05166 | HTLV-I infection | 6 |
| hsa05332 | Graft-versus-host disease | 3 |
| hsa05143 | African trypanosomiasis | 3 |
| hsa04912 | GnRH signaling pathway | 4 |
| hsa04020 | Calcium signaling pathway | 5 |
| hsa04930 | Type II diabetes mellitus | 3 |
| hsa04913 | Ovarian steroidogenesis | 3 |
| hsa04015 | Rap1 signaling pathway | 5 |
| hsa04650 | Natural killer cell mediated cytotoxicity | 4 |
| hsa05162 | Measles | 4 |
| hsa04923 | Regulation of lipolysis in adipocytes | 3 |
| hsa00140 | Steroid hormone biosynthesis | 3 |
| hsa04310 | Wnt signaling pathway | 4 |
| hsa00590 | Arachidonic acid metabolism | 3 |
